# A Bibliometric Analysis of Global Research Trends on Suicidal Ideation

**DOI:** 10.21315/mjms2023.30.4.5

**Published:** 2023-08-24

**Authors:** Nurul Hidayah Mohamad Farok, Norashikin Mahmud

**Affiliations:** School of Human Resource Development and Psychology, Faculty of Social Sciences and Humanities, Universiti Teknologi Malaysia, Johor, Malaysia

**Keywords:** bibliometric analysis, global research trends, Scopus database, suicidal ideation, VOSviewer

## Abstract

Suicide cases have increased drastically over the years, while the upsurge has inevitably spiked society’s concerns. Suicidal behaviours such as suicidal ideation have received special attention from professionals due to the harmful and irreversible consequences of possible suicide attempts. There is increasing concern that a more complete understanding of suicidal ideation trends is necessary to achieve scientific insights into suicidal behaviours through future integrated advanced research efforts. Thus, this paper aims to observe research patterns through publication outputs and co-authorships among authors and affiliated countries, besides co-occurrences of author keywords from the Scopus database. Using ‘suicidal ideation’ as the keyword on Scopus, this bibliometric analysis explored the global pattern of suicidal ideation research published between 1960 and 2020 and retrieved 3,061 records. Seven out of 15 most productive universities from the world’s top 100 best universities were found in the leading countries lists. The United States was found as dominating the research area with 80% of the publications. In conclusion, the study found that researchers have made significant progress in the research area of suicidal ideation over the years; however, the topic still warrants further analysis to understand suicidality from a broader perspective.

## Introduction

Suicidal-related behaviours have become a primary public global health concern that is increasingly affecting and threatening the mental health; psychological well-being and precious lives of existing generations, especially the vulnerable group of today’s youngsters. According to the World Health Organization (WHO) (1), suicide is the leading cause of death worldwide among individuals aged 15 years old to 29 years old. Besides, suicide rates were the highest among females aged 45 years old to 64 years old and males aged 75 years old and above (2). Based on the global statistic, suicide rates were the highest among older adults in the age group of 70 years old and above, while the lowest was among children in the age group between 5 years old and 14 years old (3).

Suicidal behaviour is a complex phenomenon, and its causes are multifactorial. Several studies among clinical adolescent populations suggested a continuum between suicidal ideation and a suicidal plan and suicide attempts (4). Thus, suicidal ideation is one of the most alarming suicidal behaviour incidents, with numerous factors affecting its occurrence. Based on Evans et al. (5), suicidal ideation is a term that broadly refers to the thoughts of committing suicidal behaviours or activities, such as self-injury or harm, regardless of a person’s intent to die. Although earlier studies had reported inconsistent findings between suicidal ideation and suicide, several later studies suggested that suicidal ideation could predict suicide attempts and suicides (6, 7).

The increasing trends of suicidal ideation among individuals have prompted researchers to further investigate and explore the primary factors leading to this highly multifactorial and contextual phenomenon. The findings of previous studies have reported that psychological factors are accounted as the main contributors of suicidal ideation experienced by individuals (8). Therefore, depression, anxiety, hostility, thwarted belongingness, physical or verbal abuse and weak social support systems have been classified among the most crucial risk factors in influencing the occurrence of suicidal ideation (9, 10). Apart from that, gender, alcohol and substance abuse and sleep quality have also been found as important factors contributing to suicidal ideation (11, 12).

Although suicidal ideation is the breeding stage of suicide-related behaviour, previous studies suggest that suicidal desires often lead to suicide attempts (13). Most victims of suicide attempts have already experienced prolonged suicidal thoughts prior to an attempt (10). A study conducted by Bryan et al. (14) concluded that repeated suicide-related thoughts provide individuals with extensively outlined suicidal plans, regardless of the intention of committing the acts, which may be regarded as a vulnerability for an attempt. Thus, this stage of suicide-related behaviours should be considered the most critical phase in seeking a professional’s help to improve an individual’s emotional well-being and daily functioning (15).

Further studies and advanced analyses are required to understand the diverse negative significant impacts of suicidal ideation on individuals’ well-being and surrounding society. Such studies will provide great insights and guidance towards understanding the occurrence of suicidal ideation and the effects on scholars and professionals related to the fields of psychiatry and psychology. Although research on suicidal ideation has gained popularity worldwide, only one bibliometric study on suicide has been conducted to determine the trends of suicide in general (16). A bibliometric analysis of suicidal ideation can help researchers understand suicidal ideation better and provide an overview of the recent trends and directions for future research on suicidal ideation. Due to this reason, this study aimed to analyse the growth of publications and journal productivity, followed by the citations, leading countries and institutions, international collaboration, prolific authors and the visualisation of common keywords in suicidal ideation publications in the field of psychology.

## Methods

The best method to adopt for this study was the bibliometric analysis approach, allowing the current researchers to thoroughly explore the overall global research growth and development of specific research interests or topics through literature in the database (17). This analysis method differentiates a bibliometric analysis paper from a review paper, whose primary purpose is to demonstrate the current performance and significant issues pertinent to a specific research area worthy of exploring.

### Screening Strategy

The present study aims to analyse and evaluate the scientific research and publication trends and growth of suicidal ideations between 1960 and 2020. Data mining in this study was carried out between 8 and 10 August 2021, using suicidal ideation as the main theme. An electronic database search was conducted in Scopus to identify all articles containing the term ‘suicidal ideation’ in the titles and abstracts. Table 1 shows the detailed procedures performed in each phase of the bibliometric analysis to obtain research articles on suicidal ideation in the field of psychology.

**Figure 1:**
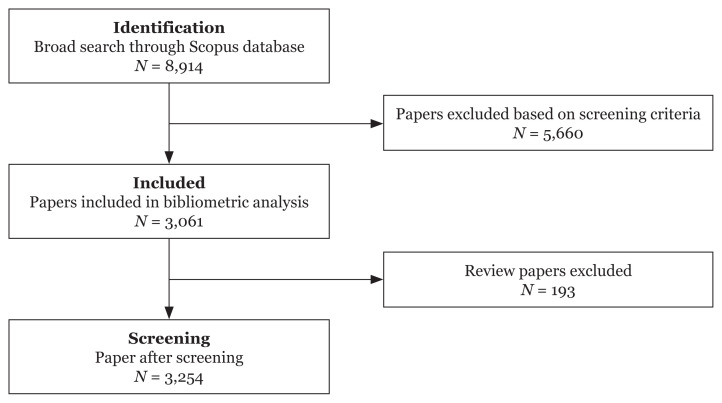
Flowchart of data collection of suicidal ideation publications in Scopus database

A total of 8,914 research articles were retrieved through the initial database search on the ‘suicidal ideation’ research topics. In the analysis, all journal articles pertaining to suicidal ideation went through a screening process involving the restrictions of certain research publications. Research articles were included only if published between 1960 and 2020 in journals within the psychology research area. Thus, papers published in peer-reviewed journals, conference proceedings and book chapters were excluded from this bibliometric analysis. Based on the screening criteria in the Scopus database’s initial search, a total of 5,660 articles were excluded. In addition, a total of 193 systematic review papers from sources deemed irrelevant for the study’s analysis were also removed. According to the screening criteria, 5,853 papers were excluded because they did not focus on the psychology research area or were published in the excluded source types. These criteria led to 3,061 finalised research articles for the bibliometric analysis. The data collection of publications on suicidal ideation is shown in Figure 1.

### Bibliometric Maps

The citation, bibliographical and author keywords from the 3,061 articles retrieved were transported to a visualising software tool, VOSviewer version 1.6.13. VOSviewer is a software tool that assists a bibliometric analysis by mapping and portraying the data network and maps through visualisation and exploration (18). All items exported into the tools were used to create a map and visualisation for further exploration. In the analysis, the author keywords and countries of publications were computed, and the links between the pair were shown in the mapping by the interconnected variables. One or more links were found among the items, indicated by the link strength. Each link’s strength was shown on an info panel with a positive numerical value, whereby the higher the value, the stronger the link.

A co-authorship analysis through a bibliometric approach is presented by the total publications (TPs) between the two affiliated countries co-authored together. The strength of the link depicts the total strength of a single country’s co-authorship with another associate country. Also, in co-occurrence analysis, the strength of the link between author keywords represents the total number of publications based on the two keywords that have appeared together in the past publication.

## Results and Discussion

### Growth Rate of Publication

**Figure 2:**
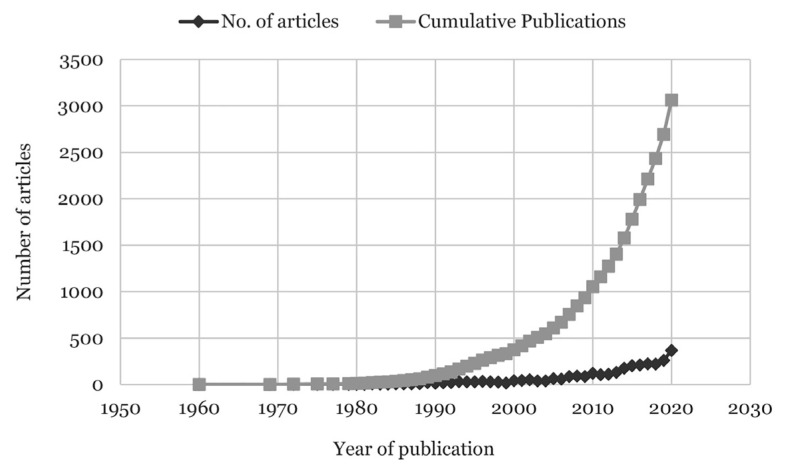
The annual and cumulative numbers of research articles on suicidal ideation retrieved from Scopus database from years 1960 to 2020

The database search in 61 years (between 1960 and 2020) yielded 3,061 published research articles on suicidal ideation. The earliest dates of publications on suicidal ideation were from Starer (19) in 1960 and no publication on this topic had been added until 1969. Research interest on suicidal ideation began to rise drastically among researchers in 1981, with an increase of more than five articles each year. From 2004, the number of publications produced escalated higher, with a minimum of 60 publications each year. By 2009, the rapid growth of research interest and suicidal ideation issues were doubled, which increased the number of annual publications and cumulative TPs. Based on this pattern, it is noteworthy that publications on this research area may continue to show steady growth and interest. The following Figure 2 shows the growth of the publication output.

### Publication Patterns

Based on the 3,061 articles retrieved from the Scopus database, only 7.1% or 216 articles were published as open access articles. The remaining 2,854 articles (92.9%) were not freely accessible, and consequently, users needed to pay to gain access to the articles’ data and information. Concerning accessibility, scholars are recommended to consider publishing articles in open-access journals to receive more citations and allow their data to be received by more professionals for further exploration regarding this topic.

Based on outputs from the database search, the results showed that articles on suicidal ideation were published using a total of 11 different languages. English was the leading language used by most authors, with 2,948 articles (96.3%) published. In addition, 57 articles (1.9%) were written in Spanish, followed by 23 articles (0.8%) in French and 13 articles (0.4%) in Portuguese. The remaining languages (30; 1.0%) included German, Italian, Japanese, Czech, Hungarian, Norwegian and Polish. To be indexed in Scopus, articles in a foreign language should contain both the title and abstract in English.

### Preferred Journals

The search outputs showed six publishers of the top 10 journals most preferred by authors in the research area, as shown in Table 1, namely Elsevier, Wiley-Blackwell, Taylors & Francis, Springer Nature, Cambridge University Press and the American Psychological Association (APA). Out of the top 10 most productive journals, the top three most preferred journals for suicidal ideation are Elsevier, Wiley-Blackwell and Taylors & Francis, as shown in Table 2.

The *Journal of Affective Disorders* was the most productive journal covering 13.9% (426 articles) of the TPs on suicidal ideation research areas, followed by second-ranked the *Suicide and Life Threatening Behaviour* (220; 7.2%), third-ranked the *Archives of Suicide Research* (154; 5.0%) and the fourth-ranked *Journal of the American Academy of Child and Adolescent Psychiatry* (106; 3.5%). The *Journal of Affective Disorders* gained the highest number of total citations (TCs) with 1,669 citations, followed by the *Suicide and Life Threatening Behaviour* with 920 TCs. The most cited suicidal ideation articles were published by APA (20) with 1,726 citations in the *Journal of Consulting and Clinical Psychology*, followed by an article with 756 times cited by Hinduja and Patchin (21), published in the *Archives of Suicide Research* by Wiley-Blackwell.

Conforming to the CiteScore 2020 report, a total of six journals were found with CiteScore of 5 and above. Table 2 shows the *Journal of the American Academy of Child and Adolescent Psychiatry*, which had the highest CiteScore of 11.1 and was ranked 4th with 106 publications in Scopus. Meanwhile, the *Journal of Clinical Psychology* with 65 publications and a CiteScore of 3.4 was ranked 9th, the lowest rank in the topmost productive journal in Scopus, which was notably lower than other journals as most of the publications (95.4%; 62 articles) were not made freely accessible, leading to its lower CiteScore.

### Leading Countries, Top Institutions and International Collaboration

**Figure 3:**
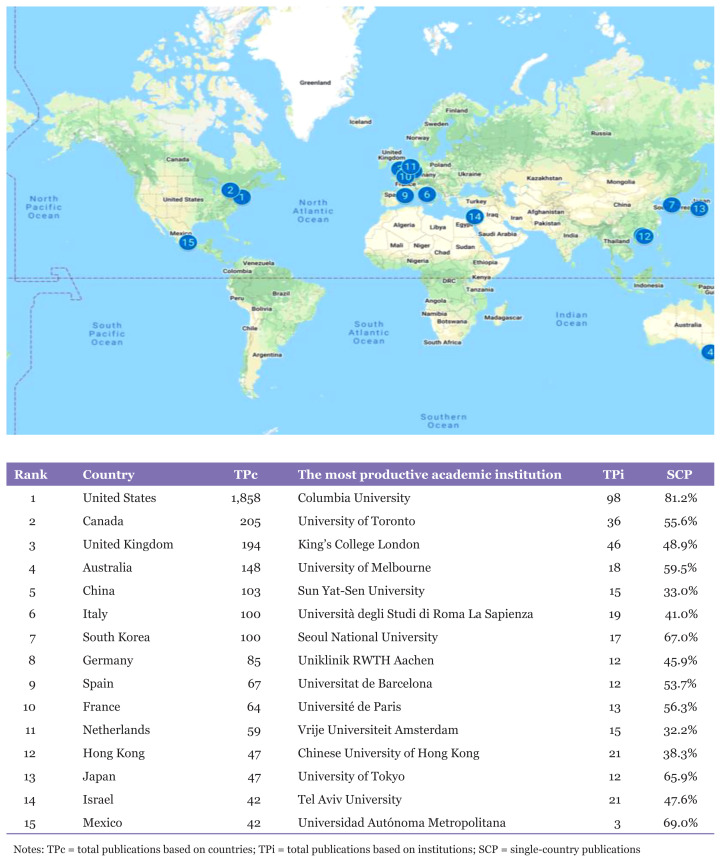
Top 15 most prolific countries and academic institutions in suicidal ideation publications

Based on the outputs, suicidal ideation publications originated from 88 countries and territories. Figure 3 shows the worldwide distributions and lists the top 15 most productive countries contributing to the trends and growth of research interest in suicidal ideation areas. United States, Canada and the United Kingdom top the list of contributing countries on the global development of the research activity on suicidal ideation, accounting for almost 74% (2,257 articles) of the total international publications. Leading the list was the United States with 1,858 publications, which involved 60.5% of the worldwide publications, followed by Canada and the United Kingdom with 205 (6.7%) and 194 (6.3%) TPs, respectively.

Along with this, 15 top academic institutions were listed according to their ranked countries, respectively. Columbia University from the United States recorded the highest TPs based on institutions (TPi) with 98 articles (3.2%); next, the University of Toronto with 36 published papers (1.2%), followed by third-ranked King’s College London with 46 publications (1.5%). The outputs indicated that this research area has governed the recognition of top academic institutions as seven leading universities listed by the World University Rankings for 100 best universities (22) were involved, namely Columbia University ranked 19th, University of Tokyo ranked 24th, University of Toronto ranked 25th, King’s College London ranked 31st, Seoul National University ranked 37th, University of Melbourne ranked 41st and Chinese University of Hong Kong ranked 43rd.

Furthermore, out of 15 countries, a total of eight countries were involved in more than half single-country publications (SCP). Ranked first, the United States had an SCP of more than two-thirds with 81.2%, followed by Mexico with 69%, South Korea with 67%, Japan with 65.9%, Australia with 59.5%, France with 56.3%, Canada with 55.6% and Spain with 53.7%. The findings indicated that countries with a high SCP steadily encouraged collaboration within the country instead of inter-country alliance or participation. Ranked 11th out of 15, the Netherlands had the lowest SCP of only 32.2%, whereby 40 out of 59 of the country’s publications were linked to multiple other international affiliations outside the country. According to Marinho et al. (23), a global alliance contributes towards healthier and more fruitful outcomes in obtaining a competitive advantage in terms of knowledge gain.

**Figure 4:**
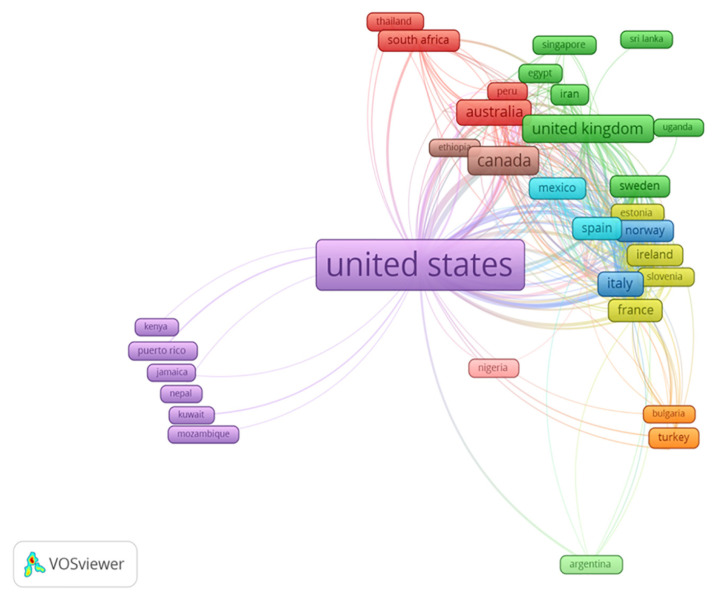
A screenshot of bibliometric map created based on co-authorships of affiliated countries

In addition to Figure 3, the countries and territories distributions per region are also illustrated in Figure 4. Based on the VOSviewer map, countries located closer to one another displayed the strength of their relatedness. In contrast, the thickness of the lines connecting them displayed the link strength among the countries. Figure 4 shows the United States as the most affiliated country linked to 64 different countries with 533 times co-authorships, followed by the United Kingdom (44 links, 205 co-authorships), Italy (41 links, 210 co-authorships), Spain (38 links, 158 co-authorships), Sweden (33 links, 117 co-authorships), Australia (33 links, 117 co-authorships) Canada (22 links, 110 co-authorships) and South Korea (14 links, 45 co-authorships).

### Leading Authors

Table 3 shows the record of the 15 most prolific authors on the central theme of suicidal ideation with all listed authors affiliated to only one country, the United States. Each author’s oldest date of publications on suicidal ideation stretched between 1964 and 2016, where each owned a respective role in co-authorship. Table 3 shows 5 out of 15 authors took a role as the first author, three as the co-author and six as the last author in their first publication, whereby no specific rules must be followed for the authorship sequence or position.

At the top of the list from the United States was Joiner TE with TP records of 55 articles on suicidal ideation since 1992, 89 h-index and a total of 30,918 TCs. Lester D from Stockton University ranked second on the list of top authors with 47 articles, 52 h-index publications and 19,839 TCs. At third-ranked was King CA with 32 TP and 43 h-index publications, while a huge gap existed in the total time cited in comparison with the previously mentioned authors, with only 5,458 TCs. It was found that authors ranked first (Joiner TE), 11th (Rogers ML) and 14th (Schmidt NB) were all affiliated to the same academic institution, Florida States University. In contrast, another pair of authors ranked fourth (Nock MK) and 15th (Kleiman EM), respectively, were both affiliated with Harvard University.

Based on Table 3, it is noted that all of the prolific authors on suicidal ideation research areas were from the United States. A possible explanation for this might be that most American authors gained significant interest in this topic as a consequence of the increasing suicide rates in the country over several decades. According to statistics, suicide is the second leading cause of death among youth in the United States, as the number of suicide cases has been increasing by 35% from 10.5 per 100,000 to 14.2 per 100,000 since 1999 (24). In comparison, the United States has consistently been ranked among other countries with the highest rate of suicide cases, including Japan, South Korea and Russia. In view of the increasing suicide prevalence rates in the United States, especially among youth, researchers are actively seeking to identify and validate various root causes and underlying mechanisms responsible for this complex phenomenon. So, it is imperative that broader scientific research into suicide-related research is carried out extensively and rigorously to curb the occurrence of suicide.

### Authors Keywords

**Figure 5:**
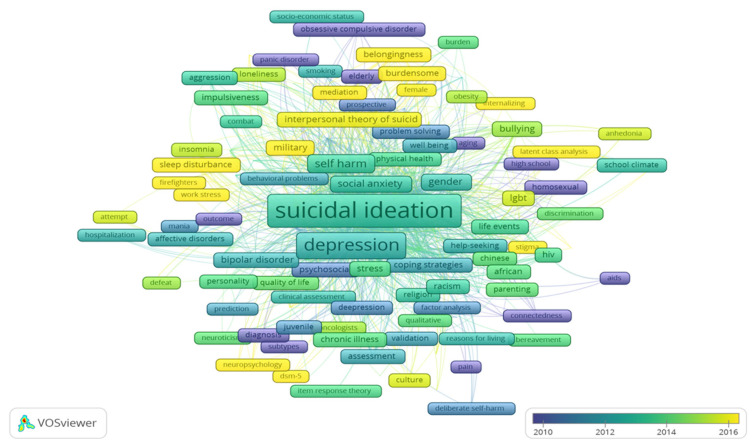
A screenshot of the bibliometric map created based on the co-occurrence of author keywords

Keywords are used by users as a search query to retrieve possible information related to their interests. In contrast, author keywords are used by authors to define the intended contents of their research publications. Figure 5 shows co-occurrences of author keywords on suicidal ideation research-related topics in the title, abstract or the full research article. The co-occurrence distribution of author keywords outlines the overview and any related research natures concerning suicidal ideation in psychology (25).

Based on the results, the most significantly highlighted keywords used by authors on this research’s nature were the general terms of ‘suicidal ideation’. The term ‘suicidal ideation’ occurred 1,539 times and was linked to 152 different other keywords. Meanwhile, other significantly used keywords in this research area include ‘depression’ (714 occurrences, 146 links), ‘adolescent’ (458 occurrences, 125 links), ‘substance abuse’ (408 occurrences, 82 links), abuse (278 occurrences, 106 links), ‘prevalence’ (242 occurrences, 99 links), ‘self-harm’ (139 occurrences, 85 links), ‘mental disorders’ (135 occurrences, 88 links), ‘well-being’ (179 occurrences, 104 links), ‘social anxiety’ (101 occurrences, 82 links) and ‘bullying’ (63 occurrences, 49 links).

## Conclusion

The bibliometric analysis on the suicidal ideation topics provided insights for scholars from various disciplines on the trend of suicidal ideation in terms of publication growth, top productive journals, countries, institutions, international collaboration and prolific authors. This study has presented extensive data on the global research trends and development of this topic based on articles information retrieved from the Scopus database. From the analyses, it was observed that the worldwide publication trend on the suicidal related topic has been increasing swiftly from 2009 onwards and is believed to continue to rise in the near future. This is due to the rapid increase in suicide rates globally, which has become a serious issue that needs extraordinary attention and thorough observation from fellow researchers, including those from disciplines related to psychology. The evidence from this study recognises the United States, Canada, Australia and the United Kingdom as the most productive countries in the world that have contributed to an enormous amount of publications on suicidal ideation areas and built a strong foundation on international collaborations to maximise insights gained from research findings. This analysis provides information on the trends of suicidal ideation research and will serve as a guide for researchers who wish to study the topic growth and development over the years.

## Limitation

The current research was limited by the term ‘suicidal ideation’ used as the initial search query string and applied to only the titles and abstracts of published articles. Thus, this process might limit the final outputs by excluding all accessible research articles interconnected to suicidal ideation related studies in the Scopus database. Some authors may have used a different term to describe suicidal ideation, such as suicidal thoughts and suicide intentions. Therefore, the search string’s first entry should be more comprehensive to gain more outputs and related articles on suicidal ideation research areas for further bibliometric analysis. It is also strongly recommended that researchers utilise multiple databases such as Web of Science, PubMed, PsycINFO and MEDLINE so that outputs from different platforms can be compared systematically through this approach.

## Recommendation

It is suggested that a systematic review should be integrated with this analysis method to obtain a deeper insight into suicidal ideation trends and the gaps that need to be addressed in suicidal ideation research. The integration of methods would give a better understanding of the research area.

## Figures and Tables

**Table 1:** The search strategies and query strings used in Scopus

Search	Query
Suicidal ideation research articles	(TITLE-ABS (“suicidal ideation”)) AND (EXCLUDE (PUBYEAR, 2021)) AND (LIMIT-TO (DOCTYPE, “ar”)) AND (LIMIT-TO (SRCTYPE, “j”))
Review articles of suicidal ideation	(TITLE-ABS (“suicidal ideation”)) AND (TITLE (“recent” OR progress OR review OR critical OR revisit OR advance OR development OR highlight OR perspective OR prospect OR trends OR bibliometric OR scientometric) OR (ABS (progress OR review OR bibliometric OR scientometric))) AND (LIMIT-TO (SRCTYPE, “j”)) AND (LIMIT-TO (DOCTYPE, “ar”)) AND (LIMIT-TO (SUBJAREA, “PSYC”)) AND (EXCLUDE (PUBYEAR, 2021))
Suicidal ideation research without review articles	(TITLE-ABS (“suicidal ideation”)) AND NOT EID (*insert EID of review articles here, see *appendix A) AND (LIMIT-TO (SRCTYPE, “j”)) AND (LIMIT-TO (DOCTYPE, “ar”)) AND (LIMIT-TO (SUBJAREA, “PSYC”)) AND (EXCLUDE (PUBYEAR,2021))

Note: EID = Scopus electronic identifier

**Table 2:** The top 10 most productive journals on psychological well-being research with their cited articles

Rank	Journal	TP (%)	TC	CiteScore 2020	The most cited article (reference)	Times cited	Publisher
1	*Journal of Affective Disorders*	426 (13.9)	1,669	6.6	The Stanley Foundation Bipolar Treatment Outcome Network - II. Demographics and illness characteristics of the first 261 patients	363	Elsevier
2	*Suicide and Life Threatening Behavior*	220 (7.2)	920	4.6	Transgender youth and life-threatening behaviours	370	Wiley-Blackwell
3	*Archives of Suicide Research*	154 (5.0)	440	4.3	Bullying, cyberbullying and suicide	756	Taylor & Francis
4	*Journal of the American Academy of Child and Adolescent Psychiatry*	106 (3.5)	744	11.1	Psychiatric risk factors for adolescent suicide: A case-control study	622	Elsevier
5	*Comprehensive Psychiatry*	85 (2.8)	257	4.9	Premorbid personality of depressive, bipolar, and schizophrenic patients with special reference to suicidal issues	201	Elsevier
6	*Depression and Anxiety*	77 (2.5)	490	9.0	Depression, desperation, and suicidal ideation in college students: Results from the American Foundation for Suicide Prevention College Screening Project at Emory University	373	Wiley-Blackwell
7	*Social Psychiatry and Psychiatric Epidemiology*	70 (2.3)	268	5.8	Suicide risk in civilian PTSD patients: Predictors of suicidal ideation, planning and attempts	132	Springer Nature
8	*Psychological Medicine*	69 (2.3)	483	9.5	Prevalence of suicide ideation and suicide attempts in nine countries	432	Cambridge University Press
9	*Journal of Clinical Psychology*	65 (2.1)	166	3.4	Suicidal ideation and help-negation: Not just hopelessness or prior help	142	Wiley-Blackwell
10	*Journal of Consulting and Clinical Psychology*	45 (1.5)	481	8.1	Assessment of suicidal intention: The scale for suicide ideation	1,726	APA

Notes: TP = total publication; TC = total citation

**Table 3:** List of 15 most prolific authors in suicidal ideation research area

Rank	Author	Scopus author ID	Year of 1st publication	TP	h-index	TC	Current affiliation	Country
1	Joiner TE	35547584300	1992^b^	55	89	30,918	Florida State University	United States
2	Lester D	36049464300	1964^b^	47	52	19,839	Stockton University	United States
3	King CA	35444841900	1981^a^	32	43	5,458	University of Michigan, Ann Arbor	United States
4	Nock MK	6701414081	1998^c^	31	76	23,986	Harvard University	United States
5	Spirito AS	7006501688	1979^c^	29	61	12,939	Brown University	United States
6	Gutierrez PM	7103382249	1996^a^	25	35	5,703	University of Colorado School of Medicine	United States
7	Miranda R	7102041744	2000^b^	22	20	1,814	Hunter College	United States
8	Conner KR	7005832037	1994^a^	21	43	5,718	University of Rochester Medical Center	United States
9	Anestis MD	15821688600	2007^c^	20	35	3,817	New Jersey Gun Violence Research Center	United States
10	Bryan CJ	21933788900	2006^a^	20	38	4,190	The University of Utah,	United States
11	Rogers ML	56523887000	2016^c^	20	17	10,99	Florida State University	United States
12	Pettit JW	7005407988	2000^c^	19	34	3,292	Florida International University	United States
13	Kaslow NJ	57214701491	1983^a^	16	53	9,349	Emory University School of Medicine	United States
14	Schmidt NB	7101860643	1990^a^	16	63	14,114	Florida State University	United States
15	Kleiman EM	37031224700	2010^c^	15	25	2,916	Harvard University	United States

Notes:

a first author;

b co-author;

c last-author;

TP = total publication; TC = total citation

## Appendix

EID of review articles:

2-s2.0-85074965852 OR 2-s2.0-85073652112 OR 2-s2.0-85057183887 OR 2-s2.0-85068908778 OR 2-s2.0-85068506972 OR 2-s2.0-85053860347 OR 2-s2.0-85053073425 OR 2-s2.0-85061792428 OR 2-s2.0-85066792251 OR 2-s2.0-85055782177 OR 2-s2.0-85054606930 OR 2-s2.0-85046896187 OR 2-s2.0-85063603717 OR 2-s2.0-85041580907 OR 2-s2.0-85060288504 OR 2-s2.0-85063083960 OR 2-s2.0-85056653010 OR 2-s2.0-85022207145 OR 2-s2.0-85057539157 OR 2-s2.0-85072345574 OR 2-s2.0-85072314162 OR 2-s2.0-85072305463 OR 2-s2.0-85068724969 OR 2-s2.0-85054924949 OR 2-s2.0-85069482532 OR 2-s2.0-85077923600 OR 2-s2.0-85067403986 OR 2-s2.0-85059245719 OR 2-s2.0-85048181244 OR 2-s2.0-85048556870 OR 2-s2.0-85048032404 OR 2-s2.0-85051400206 OR 2-s2.0-85047636435 OR 2-s2.0-85039802233 OR 2-s2.0-85054588568 OR 2-s2.0-85051081828 OR 2-s2.0-85051079280 OR 2-s2.0-85051064175 OR 2-s2.0-85045453292 OR 2-s2.0-85050450819 OR 2-s2.0-85056993870 OR 2-s2.0-85030454648 OR 2-s2.0-85015413258 OR 2-s2.0-85017586221 OR 2-s2.0-85037633791 OR 2-s2.0-85056418345 OR 2-s2.0-85044633948 OR 2-s2.0-85034867699 OR 2-s2.0-85032305214 OR 2-s2.0-85034111377 OR 2-s2.0-85031778791 OR 2-s2.0-85022084231 OR 2-s2.0-85018809055 OR 2-s2.0-85027564963 OR 2-s2.0-85043382778 OR 2-s2.0-85012306136 OR 2-s2.0-85015639845 OR 2-s2.0-84927556659 OR 2-s2.0-84986210474 OR 2-s2.0-85028421009 OR 2-s2.0-84953911539 OR 2-s2.0-85007320978 OR 2-s2.0-84949009638 OR 2-s2.0-85040987277 OR 2-s2.0-84975717676 OR 2-s2.0-85020546540 OR 2-s2.0-84999018453 OR 2-s2.0-85001736044 OR 2-s2.0-84989887514 OR 2-s2.0-84994891150 OR 2-s2.0-84994449199 OR 2-s2.0-84983078658 OR 2-s2.0-84973450437 OR 2-s2.0-84955568273 OR 2-s2.0-84959054518 OR 2-s2.0-84953230713 OR 2-s2.0-84964264786 OR 2-s2.0-84958174198 OR 2-s2.0-84959536558 OR 2-s2.0-84964547544 OR 2-s2.0-84959128298 OR 2-s2.0-84961262910 OR 2-s2.0-84941348915 OR 2-s2.0-84947613459 OR 2-s2.0-84992710986 OR 2-s2.0-84894280416 OR 2-s2.0-84911374388 OR 2-s2.0-84982132695 OR 2-s2.0-84943456895 OR 2-s2.0-84930759559 OR 2-s2.0-84943453769 OR 2-s2.0-84920855523 OR 2-s2.0-84938416418 OR 2-s2.0-84948102844 OR 2-s2.0-84936949132 OR 2-s2.0-84923816383 OR 2-s2.0-84930178498 OR 2-s2.0-84900426449 OR 2-s2.0-84889085294 OR 2-s2.0-84904604975 OR 2-s2.0-84902475273 OR 2-s2.0-84929312826 OR 2-s2.0-84897472680 OR 2-s2.0-84927959943 OR 2-s2.0-84902306419 OR 2-s2.0-84939872325 OR 2-s2.0-84897804873 OR 2-s2.0-84918585233 OR 2-s2.0-84881024240 OR 2-s2.0-84878494502 OR 2-s2.0-84880938694 OR 2-s2.0-84883105083 OR 2-s2.0-84882572567 OR 2-s2.0-85027942199 OR 2-s2.0-84869164991 OR 2-s2.0-84866662116 OR 2-s2.0-84860582218 OR 2-s2.0-84855573106 OR 2-s2.0-84855533989 OR 2-s2.0-84856006933 OR 2-s2.0-84863212946 OR 2-s2.0-84864236278 OR 2-s2.0-80053453754 OR 2-s2.0-80054959937 OR 2-s2.0-84862873165 OR 2-s2.0-81255172821 OR 2-s2.0-79953225840 OR 2-s2.0-79954463046 OR 2-s2.0-79851513709 OR 2-s2.0-79951846017 OR 2-s2.0-80052472538 OR 2-s2.0-78751661136 OR 2-s2.0-77957989107 OR 2-s2.0-77949656073 OR 2-s2.0-75749140496 OR 2-s2.0-71649102804 OR 2-s2.0-77951661010 OR 2-s2.0-76849093178 OR 2-s2.0-72949096795 OR 2-s2.0-70449596102 OR 2-s2.0-63349107543 OR 2-s2.0-65549116322 OR 2-s2.0-42649132100 OR 2-s2.0-45849083118 OR 2-s2.0-36048986715 OR 2-s2.0-34548059867 OR 2-s2.0-41849115980 OR 2-s2.0-36549058831 OR 2-s2.0-33947642008 OR 2-s2.0-33845756248 OR 2-s2.0-33947221517 OR 2-s2.0-33845382298 OR 2-s2.0-32844470930 OR 2-s2.0-33845890806 OR 2-s2.0-28644451968 OR 2-s2.0-20444486489 OR 2-s2.0-20444454347 OR 2-s2.0-8744260621 OR 2-s2.0-4344712110 OR 2-s2.0-0037301637 OR 2-s2.0-0642311899 OR 2-s2.0-0041968809 OR 2-s2.0-0345304812 OR 2-s2.0-0037495042 OR 2-s2.0-0036003736 OR 2-s2.0-0036389180 OR 2-s2.0-0035459055 OR 2-s2.0-0035676462 OR 2-s2.0-0034991357 OR 2-s2.0-0033448760 OR 2-s2.0-0032134814 OR 2-s2.0-0032142828 OR 2-s2.0-0032328764 OR 2-s2.0-0031741739 OR 2-s2.0-0031023649 OR 2-s2.0-0029940533 OR 2-s2.0-0030271321 OR 2-s2.0-0029560043 OR 2-s2.0-0029128201 OR 2-s2.0-0028807181 OR 2-s2.0-0028070181 OR 2-s2.0-0028086490 OR 2-s2.0-84965404618 OR 2-s2.0-0025913454 OR 2-s2.0-0025835755 OR 2-s2.0-0000711997 OR 2-s2.0-0025241752 OR 2-s2.0-0024839628 OR 2-s2.0-0021853499 OR 2-s2.0-0016358568 OR 2-s2.0-85052848203 OR 2-s2.0-85032272008 OR 2-s2.0-84903194101
